# Articular Disc of a Human Temporomandibular Joint: Evaluation through Light Microscopy, Immunofluorescence and Scanning Electron Microscopy

**DOI:** 10.3390/jfmk6010022

**Published:** 2021-02-25

**Authors:** Michele Runci Anastasi, Piero Cascone, Giuseppe Pio Anastasi, Giuseppe Santoro, Fabiana Nicita, Giacomo Picciolo, Angelo Favaloro, Giuseppina Rizzo, Giuseppina Cutroneo

**Affiliations:** 1 IRCCS (Istituto di Ricovero e Cura a Carattere Scientifico) Centro Neurolesi “Bonino-Pulejo”, 98100 Messina, Italy; michele.runci@gmail.com; 2 Department of Maxillo-Facial Surgery, University of Roma, La Sapienza, 00185 Roma, Italy; piero.cascone@uniroma1.it; 3 Department of Biomedical and Dental Sciences and Morphofunctional Imaging, University of Messina, 98100 Messina, Italy; anapuc@unime.it (G.P.A.); giuseppe.santoro@unime.it (G.S.); gpicciolo@unime.it (G.P.); afavaloro@unime.it (A.F.); giuseppina.rizzo@unime.it (G.R.); 4 Department of Clinic and Experimental Medicine, University of Messina, 98100 Messina, Italy; fabin92@hotmail.it

**Keywords:** temporomandibular joint, articular disc, collagen, elastin

## Abstract

The extracellular matrix of the articular disc in a temporomandibular joint (TMJ) is composed mainly of collagen I and elastin. The collagen is important for resisting tensile forces, while the elastin is responsible to maintain the shape after deformation. We studied the orientation of collagen and elastin in a normal human temporomandibular joint disc by light microscopy, immunofluorescence and scanning electron microscopy. Our results demonstrated that collagen and elastin run parallel to each other in the intermediate zone with an anteroposterior orientation. From here, the orientation of two fibers groups changes into a disordered arrangement in the transition zone. Numerous elastic fibers cross with the collagen fibers, defining an interwoven knitted arrangement. The evaluation of the disc–condyle relationship shows that the medial margin of the articular disc is inserted directly at the superficial layer of the mandibular condylar cartilage. Therefore, the tensile properties of the TMJ disc are expressed in the directions corresponding to the orientation of the collagen fibers, and the complex orientation of elastin with the collagen determines the maintaining of the shape after the stresses by the joint movements. Moreover, the direct anatomical relationship between the articular disc and the mandibular condyle makes a decisive contribution to the understanding of TMJ movements.

## 1. Introduction

The temporomandibular joint’s articular disc is a biconcave structure made up of fibrocartilage tissue; it can be divided into three different functional portions: posterior band, intermediate zone and anterior band [1,2,3]. The thin intermediate zone allows flexibility, enables smooth articulation and protects the superior and inferior articulating surfaces. The thick anterior and posterior bands fill the space created by the convex surface of the mandibular condyle and also provide structural integrity to the disc [4,5,6,7].

The extracellular matrix of the disc is composed mainly of collagen type I and elastin, with small amounts of collagen II and trace amounts of type III, VI, IX and XII [8,9]. The collagen I type is the predominant component, forming a great oblique and lateral network, and it is very important for resisting tensile forces [10,11]. The arrangement and orientation of collagen fibers varies by location in the disc and in pathological condition [12]. The intermediate zone has large fibers generally oriented parallel to the surfaces of the disc in the anteroposterior direction. These fibers turn mediolaterally and supero-inferiorly with the adjacent fibers [13,14,15]. Fibers groups with mainly transverse orientation are confined within the anterior and posterior bands. When the intermediate zone fibers reach the anterior and posterior bands, they widen superiorly and inferiorly, and some of them become continuous, with transversely oriented fasciculi. At the medial and lateral disc margins, some of the transversely oriented fascicule of the bands are confluent with the anteroposteriorly oriented fibers [16]. These combined fibers form a ringlike structure around the periphery of the disc [11,17].

The elastin is associated with resistance and elasticity and is responsible for maintaining the shape after deformation. Its distribution is different for regions. The superior layer of the disc contains more elastic fibers than the inferior layer, and their number increases from the center toward the margins of the disc [18,19].

Elastic fibers generally run along, in between and parallel to the collagen fibers and may branch, forming a network [20,21,22,23].

The relationships of collagen and elastic fiber groups within the disc are complex, and a number of ambiguities remain with respect to their regional variations. These connections are important to the behavior of the articular disc in the normal joint. The aim of this study is to evaluate the behavior and orientation of collagen type I and elastin on the different portions of the articular disc of the human temporomandibular joint (TMJ) and the comparison of them under light microscopy, immunofluorescence and scanning electron microscopy (SEM). Finally, it is still not clear how the simultaneous movements between disc and condyle can occur and if there are anatomical connections that can play a crucial role in TMJ function. Therefore, we analyzed the medial and lateral margins of the disc through serial images by light microscopy.

## 2. Materials and Methods

### 2.1. Samples

For the study, we used 9 heads of human cadavers (6 male and 3 female, 65 +/− 3 years old), preserved in the anatomical museum of the Department of Biomedical and Dental Sciences and Morphofunctional Images of University of Messina. The anatomical samples of left and right TMJ were selected according to the SCAPINO criteria [24]: smooth articular surfaces, articular disc without perforations, its posterior band situated above or only slightly anterior to the summit of the condyle in all regions and no signs of disc displacement or temporomandibular disorder. The macro-anatomical dissection of the specimens was carried out in sagittal planes with the vision of the articular eminence, TMJ disc in the anteroposterior view, condylar bone portion and retro-discal tissue. For each articular disc sample, the intermediate zone, the anterior and posterior bands and the transition zone were studied. We mean the “transition zone” as a region between the intermediate zone and the bands. Then, we analyzed these regions with three different techniques (light microscopy, immunofluorescence and scanning electron microscopy (SEM)) to study the distribution and orientation of the main components of the articular disc. Subsequently, we analyzed the macro-anatomical dissections of the samples on the coronal plane. We have obtained serial histological sections of the lateral and medial sides of the articular disc in the anteroposterior direction.

### 2.2. Light Microscopy

The specimens were fixed overnight in 4% paraformaldehyde in 0.05-M phosphate buffer at 4 °C, dehydrated in ethanol and infiltrated with Technovit9100. Sections of 7-μm thickness were cut with a microtome Leica RM 2125RT (Leica Biosystem, Nussloch, Germany) and were stained by Mallory’s trichrome method with carbolfuchsin for 15 min, acid buffer for 2 min and phosphomolybdic acid for 5 min and with the hematoxylin and eosin method. The sections were examined and photographed with a light microscope Eclipse Ci-L (Nikon Corporation, Tokyo, Japan).

### 2.3. Immunofluorescence

The biopsies were fixed in 3% of paraformaldehyde in 0.2-M phosphate buffer, pH 4.4, for 2 h at room temperature. They were washed extensively with 0.2-M phosphate buffer, pH 7.4, and then with phosphate-buffered saline (PBS) containing 12% and 18% sucrose. The samples were snap-frozen in liquid nitrogen, and 20-μm sections were prepared in a cryostat for use in a protocol to perform immunofluorescence. The sections were placed on glass slides that were coated with 0.5% gelatin and 0.005 chromium potassium sulfate. To block nonspecific binding sites and to permeabilize the membranes, the sections were preincubated with 1% bovine serum albumin (BSA) and 0.3% triton X-100 in PBS for 15 min at room temperature. Finally, the sections were incubated with primary antibodies. We used the following primary monoclonal mouse antibodies: anti-collagen type I (Sigma Aldrich, St. Louis, MO, USA), dilution range 1:1000, and anti-elastin (Santa Cruz Biotechnology Inc., Santa Cruz, CA, USA), dilution range 1:50.

Primary antibodies were detected using Texas-Red-conjugated immunoglobulin (IgG) anti-mouse (Jackson ImmunoReseach Laboratories, Inc., West Grove, PA, USA) and Fluorescein Isothiocyanate (FITC) (Jackson ImmunoReseach, Inc., West Grove, PA, USA), both at a dilution range of 1:100 [25,26,27].

The sections were analyzed and images acquired using a Zeiss LSM 5 Duo (Carl Zeiss, Iena, Germany) confocal laser scanning microscope. All images were digitalized at the resolution of 8 bits into an array of 2048 × 2048 pixels. Optical sections of fluorescence specimens were obtained using a HeNe laser (wavelength = 543 nm) and an Ar laser (wavelength = 458 nm) at a 762-s scanning speed with up to 8 averages; 1.50-μm-thick sections of fluorescence specimens were obtained using a pinhole of 250. Contrast and brightness were established by examining the most brightly labeled pixels and choosing the settings that allowed a clear visualization of the structural details while keeping the pixel intensity at its highest (200). Each image was acquired within 62 s in order to minimize the photodegradation. For image analysis, we used the function called “splitting”, showing individual channels and relative merges and the “acquisition of a z-stack” to analyze the staining pattern in the entire thickness of the section. We also applied a function called “3-dimensional depth code” showing the depth information in fluorescence and closed in the stack of a section. Digital images were cropped and figure montages were prepared using Adobe Photoshop 7.0 (Adobe System, Palo Alto, CA, USA).

A semiquantitative analysis was performed by an observer based on the color intensity of collagen type I and elastin both for the light microscopy method and the immunohistochemical one [28,29,30].

### 2.4. Scanning Electron Microscopy (SEM)

Biopsies were fixed in 2.5% glutaraldehyde in 0.1-mol/L phosphate buffer, pH 7.4, for 24 h at room temperature. Samples were dehydrated with ethanol and amyl acetate and critical point-dried through liquid CO_2_. The fractured surface of bone was then placed on a stub, coated in a Plasma Science CrC Turbo Pumped (Torr International, New Windsor, NY, USA) and was observed with a Phenom G2 scanning electron microscope (Phenom, Eindhoven, The Netherlands) [31,32,33].

## 3. Results

From each sample of TMJ, obtained from the sections in the sagittal and coronal planes, we observed the articular eminence, disc, condylar bone portion and retro-discal tissue. In all the specimens, the articular surfaces were smooth, the articular disc was without perforations and there were no signs of disc displacement or temporomandibular disorder. For each anatomical sample, we considered the articular disc and analyzed the four regions (intermediate zone, anterior and posterior bands and transition zone) through light microscopy, immunofluorescence and SEM and the lateral and medial sides of the disc by means of light microscopy and immunofluorescence.

### 3.1. Light Microscopy and Immunofluorescence

**Figure 1:**
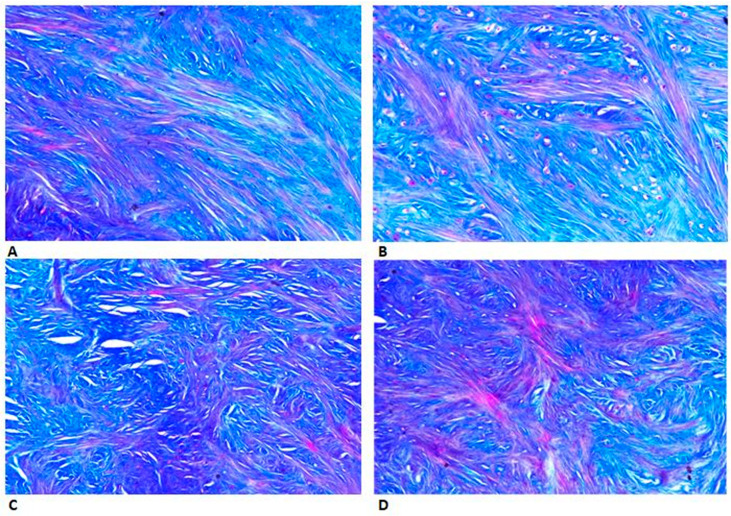
Distribution of collagen (blue color) in the articular disc sections performed with light microscopy. (**A**) Collagen has a parallel orientation in the intermediate zone. (**B**) Collagen passing from the parallel arrangement of the intermediate zone to an intersection orientation (transition zone). (**C**) Interwoven knitted arrangement of collagen fibers in the anterior band. (**D**) Bundles of collagen fibers crossing each other with a knitted orientation in the posterior band. Staining with Azan-Mallory (20×).

Analyzing the sections performed with light microscopy, we found that collagen fibers have a parallel orientation in the intermediate zone (Figure 1A,B). The collagen is more represented in the transition zone, and it is visible as fibers groups pass from the parallel arrangement of the intermediate zone to an intersection orientation (Figure 1C,D). To visualize the distribution of collagen and elastin in the articular disc, and to study the staining pattern in relation to the optical field, we performed immunofluorescence.

**Figure 2:**
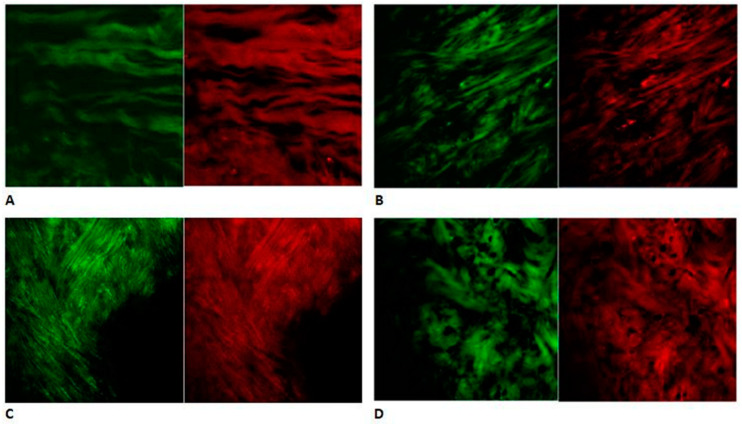
Distribution of collagen and elastin in the articular disc sections performed with the immunofluorescence method. (**A**) Collagen (green channel) and elastic fibers (red channel) have a wavy longitudinal and parallel orientation and superimposable fluorescence pattern in the intermediate zone. (**B**) The green fluorescence pattern of collagen is more marked, and the collagen and elastic fiber group orientations change to a disordered arrangement in the transition zone. (**C**) The red pattern of elastin is more represented, and the bundles of collagen and elastic fibers have a knitted arrangement in the anterior band. (**D**) The elastin pattern (red channel) is more marked, and the collagen (green channel) and elastic fibers have a knitted orientation in the posterior band (20×).

In the intermediate zone, the collagen and elastic fibers have a wavy longitudinal and parallel orientation and superimposable fluorescence pattern (Figure 2A). In the transition zone section, the fluorescence pattern of the collagen is the main component, and the two fiber group orientations change to a disordered arrangement (Figure 2B). In the anterior and posterior bands, the elastin pattern is more represented, and the bundles of collagen and elastic fibers cross each other with a knitted arrangement (Figure 2C,D).

**Figure 3:**
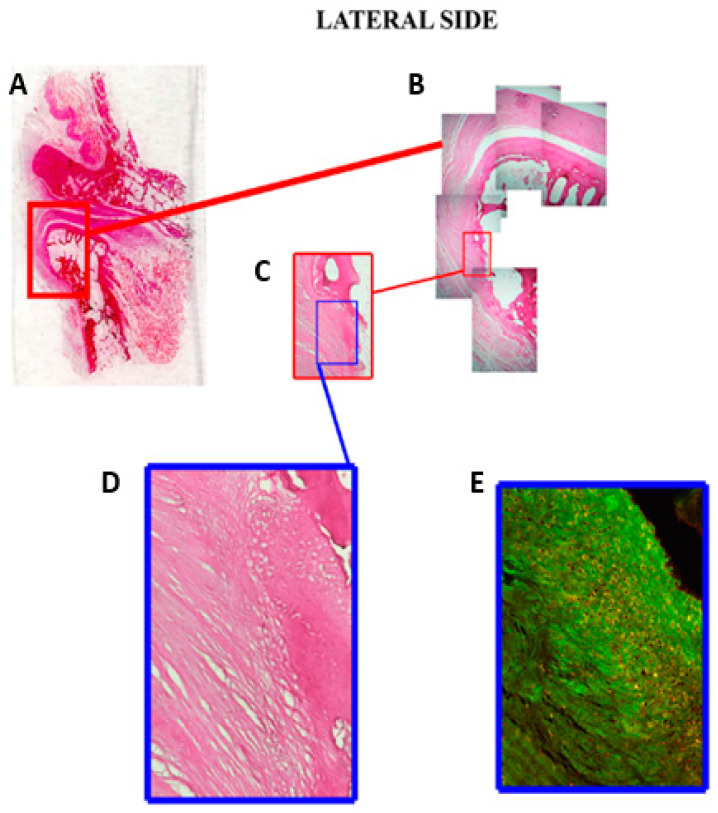
Coronal sections of the temporomandibular joint (TMJ) stained with hematoxylin and eosin. (**A**) Direct photography of glass with this section shows the lateral insertion of the disc on the articular surface of the condyle. (**B**) The reconstruction with micrographs shows the structural characteristics of the disc and the articular surface of the condyle (10×). The direct insertion of the collagen fibers of the disc in the articular cartilage is better shown in (**C**) (20×) and (**D**) (40×). (**E**) The corresponding section treated with immunofluorescence (double-localization), using anti-collagen type I (green channel) and anti-elastin (red channel), shows the prevalence of collagen fibers in the region of the insertion (40×).

The evaluation of the disc on the coronal plane in the anteroposterior direction shows that the lateral margin of the articular disc is inserted on the articular surface of the condyle (Figure 3A,B). The magnified histological section shows that the collagen fibers of the disc are inserted into the articular cartilage (Figure 3C,D). The immunofluorescence image, corresponding to the histological section described above, highlights the prevalence and orientation of collagen fibers towards the articular cartilage of the condyle (Figure 3E).

**Figure 4:**
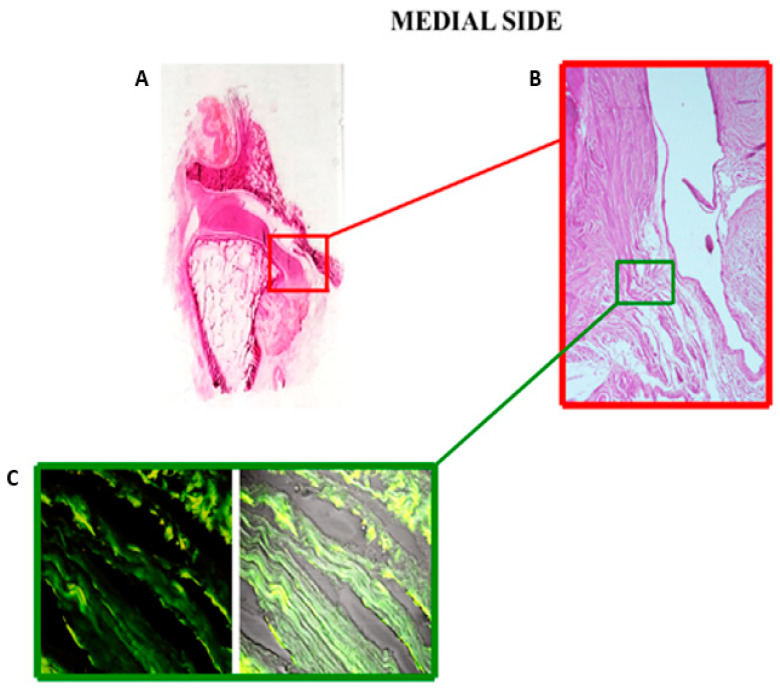
Coronal sections of the TMJ stained with hematoxylin and eosin. (**A**) Direct photography of glass with this section shows the medial insertion of the disc on the articular surface of the condyle. (**B**) The micrograph shows the insertion of collagen fibers in the medial region of the articular surface (20×). (**C**) The double-localization using anti-collagen type I (green channel) and anti-elastin (red channel) shows the almost exclusive prevalence of collagen fibers in the insertion site (40×).

In the medial portion of the TMJ, the disc makes a loop, continues and connects directly with the most superficial layer of the articular cartilage at the postero-upper part of the condylar pole (Figure 4A,B). The magnified histological image shows that the collagen fibers of the disc extend into the fibrous cartilage to become strongly thickened fibers approximately parallel to the articular surface. The immunofluorescence section of the double-localization reaction highlights the insertion of the collagen fibers of the articular disc directly into the fibrous cartilage (Figure 4C).

### 3.2. SEM

**Figure 5:**
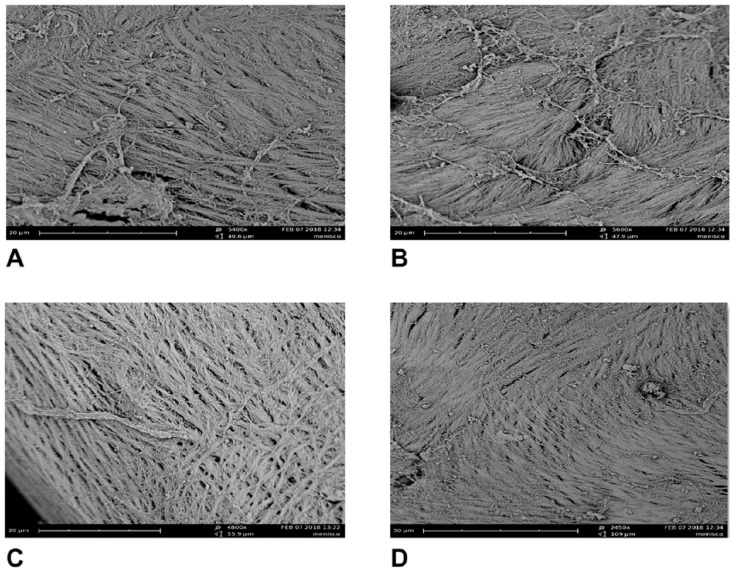
Arrangement of fiber components of the articular disc performed with scanning electron microscopy (SEM). (**A**) In the intermediate zone, compact fibers bundles run parallel to each other in the anteroposterior direction (5400×). (**B**) Many fibers of the intermediate zone become branched and disorganized from running in different directions in the transition zone (5600×). (**C**) Compact fiber bundles with sagittal or oblique orientations, and which intermingle with fibers of the transverse orientation, were found in the anterior band (4800×). (**D**) Large network structures of dense bundles were arranged anteroposteriorly, transversally and obliquely in the posterior band (2450×).

The SEM scans confirmed the results of the fiber arrangements obtained from the previous methods for the three disc regions in sagittal vision. In the intermediate zone, fibers groups are represented by compact bundles and run parallel to each other in the anteroposterior direction (Figure 5A). From there, most of the fibers extend into the regions of the disc bands, where the fiber bundles adjacent to the disc surfaces begin to flare in different directions (Figure 5B). In the anterior band, there are compact bundles of collagen fibers with sagittal or oblique orientations that intermingle with fibers in the transverse orientation (Figure 5C).

Large network structures of collagen-dense bundles with numerous fibers arranged in a complex fashion are found in the posterior band. The fibers in these bundles are arranged anteroposteriorly, transversally and obliquely. These network structures surround transversely oriented collagen bundles. Numerous elastic fibers cross with the collagen fibers network (Figure 5D).

## 4. Discussion

Despite that human and porcine TMJ discs are considered similar in size, shape, joint anatomy and masticatory pattern [34,35,36], the human TMJ disc behaves anisotropically, with differences in the principal fiber orientations and organization in each region. We studied the distribution and the arrangement of collagen type I and elastin in terms of their contributions to the TMJ mechanics.

Many authors have described the arrangement of collagen fibers in the human TMJ disc. Collagen fibers, arranged in a sagittal direction in the anterior and middle portions of the disc, were described by Castellaneta [37] and Griffin et al. [38]. However, the fibers assume other directions in the posterior portion [39] and exhibit a network-like nature in the anterior and posterior portions [40]. Elastic fibers are small in number in the disc and are important in restoring and retaining the resting disc form and position after loading [19,41]. Moreover, these fibers do not contribute sufficiently to the tensile strength [39] and provide little resistance to elongation [13].

In this study, we used light microscopy as the first method to evaluate collagen type I and the elastin orientation in four different disc regions (intermediate zone, transition zone and anterior and posterior band). The results of this technique demonstrated that the two fibers groups have a parallel orientation in the intermediate zone and an interwoven knitted arrangement in the band regions. These results are in contrast with previous research that stated that the elastic fibers lie parallel to the collagen fibers in the three portions of the disc [11,23,32,33,34,35,36,42]. Moreover, there is a change of orientation of the two fibers bundles in the transition zone. In particular, we observed that the fibers pass from a parallel arrangement to a crossover orientation. These evaluations were confirmed by the support of immunofluorescence, which further highlighted the arrangement of the collagen and elastic fibers. In this regard, the transition zone section clearly showed the change in orientation of the fibers groups in question. This result was innovative, because in the literature, there are still no studies that describe this transition zone as a passage between the intermediate zone and the posterior or anterior bands. Only the study of Minarelli et al. [11] reveals a disc/capsule transition zone with a few elastic fibers lying perpendicular to the collagen fibers.

Light microscopy and immunofluorescence scans performed a semiquantitative analysis of the collagen and elastin in the analyzed regions. Collagen is the main component in the transition zone, while the elastin is represented more in the intermediate zone and band regions.

Although it is believed that collagen correlates with tensile properties, in reality, the tensile strength and stiffness of the disc depend more on the local orientation of collagen than on its total contents [36,43]. Instead, the elastin content likely does not manifest itself in traditional mechanical properties but, rather, may serve as an indicator of the disc regions more prone to stretching and recovery during jaw movement [8]. SEM performed a nondestructive investigation and allowed us to obtain morphological and structural information on the collagen and elastic fibers in the sections considered. In the intermediate zone, collagen fibers ran predominately in the anteroposterior direction and were tightly bound. The elastic fibers ran parallel to the collagen bundles, supporting the primary direction of stretching in the anteroposterior direction. When the intermediate zone transitioned to the bands, the collagen and elastic fibers ran continuously with those in the bands, but they became branched and disorganized. In the posterior band, dense bundles of collagen were arranged anteroposteriorly, transversally and obliquely and surrounded transversely oriented collagen bundles. Accordingly, the fibers ran circumferentially along the periphery, enclosing the intermediate zone and forming a ring-like structure. Compact bundles of collagen fibers with sagittal or oblique orientations, and that intermingled with fibers of transverse orientation, were found in the anterior band. A few elastic fibers were found around the collagen bundles in the anterior band, while numerous elastic fibers were concentrated in the posterior band. We can make predictions from our results about how the disc will behave during joint movement. The collagen arrangement is reflected in the higher tensile stiffness and strength to radial stresses in the intermediate zone and circumferential stresses in the bands. We found that some collagen fibers aligned perpendicularly or obliquely in relation to the disc surface in the bands. These different orientations of collagen fibers that intertwined with each other formed a complex network. This would confirm the importance of collagen anisotropy in the mechanical properties of the TMJ.

The distribution of elastin in the disc is controversial and has always been described as irregular [23]. In this study, the marked red fluorescence pattern in the posterior band can be explained in relation to the insertion of the upper bilaminar zone, which contains thick and abundant elastic fibers [24]. In fact the upper layer of the bilaminar zone provides an elastic force to pull the disc from the anterior position to the resting position on top of the condyle during the mandible opening and closing [44,45]. Some elastic fibers are concentrated in the anterior portion, due to the relationship with the articular capsule. The elastin pattern is superimposable, and its parallel orientation with collagen in the intermediate zone suggests that this area is more exposed to a great load and stress during TMJ opening and closing movements.

Another important objective proposed by our research was to analyze the margins of the articular disc through serial images of light microscopy. In this regard, the literature reports conflicting results and considerations. An overview of 2013 [46] considered that the disc is attached to the fibrous capsule of the joint not only anteriorly and posteriorly but, also, medially and laterally, which divides the TMJ into two distinct cavities. The histological investigation of Willard et al. [47] on porcine TMJ discs mentioned disc attachments and stated that the medial and lateral attachments join mainly with the inferior mandibular condyle. The disc blends into the medial attachment high in the joint, and the attachment to the condyle is at the top of the condylar head, whilst the lateral attachment connects very low on the condylar neck. Our serial images of light microscopy revealed that the articular disc is inserted at the articular cartilage of the mandibular condyle on the lateral side. Analyzing the histological and immunofluorescence sections corresponding to the magnification of the disc insertion in the cartilage, it is evident that the collagen fibers of the disc join with those of the articular surface of the condyle. However, the articular disc, on the medial side, connects directly with the most superficial layer of the articular cartilage at the postero-upper part of the condylar pole. The histological section corresponds to the magnification of the disc insertion in the cartilage. Our results could provide a hypothesis that the disc is firmly coupled to the mandibular condyle, allowing simultaneous joint movements. Even a radiological study [48] highlighted that the disc is not attached to the joint capsule medially and laterally, unlike its anterior and posterior attachments. Instead, the disc is firmly attached to the medial and lateral poles of the mandibular condyle. The direct insertion of the disc in the articular cartilage could be explained by an embryological point. Condylar cartilage is distinctively different in composition and mechanics compared to hyaline cartilages, knee meniscus or the growth plate [49]. The fibrocartilage found in the TMJ is a secondary cartilage [50]. Secondary cartilages develop from undifferentiated cells comprising mesenchymal tissue covering the prenatal or postnatal condyle, while primary cartilage (found in all other articulations) growth begins in the cartilage cells within the central layer of an epiphyseal plate [51]. From a biomechanical point of view, the mandibular condyle cartilage withstands a large amount of occlusal force placed on the TMJ [52]. Moreover, this cartilage has other advantages: fibers are tightly packed to support the forces of movement; they are less likely to break down over time and have a better ability to repair themselves.

Our findings are important for establishing the biomechanical response of the human TMJ disc and the structure–function relationships between the collagen and elastic fiber directions and organizations.

In conclusion, collagen distribution in the TMJ disc is anisotropic, with a directional alignment rather than a random orientation. Such an arrangement generates high tensile properties in directions corresponding to the fiber alignment. The formation of ringlike collagen organization in the bands surrounding the intermediate zone is critical for the disc’s role in enduring the radial and circumferential stresses in the rotation and translation. Elastin is present throughout the disc, with regional variations in the distribution and organization. Its complex and intertwined orientations in the bands and the parallelism with the collagen fibers in the intermediate zone contribute to the recovery of the disc tissues after the deformation determined by the joint movements. Moreover, the thick bands of the disc, placed around the intermediate zone, automatically tend to keep the disc in the correct position on the articular surface of the condyle. When the condyle rotates, resistance is created to the slipping between the condyle and the disc, so that the disc can passively follow the movements of the mandibular condyle. We demonstrated a tight anatomical anchoring of the disc to the condylar head by means of serial histological images. In addition to the self-positioning property of the disc, the direct insertions of the disc in the condylar periosteum of the lateral side and in the mandibular cartilage of the medial side could facilitate simultaneous movements of the TMJ disc–condyle.

## Data Availability

The data that support the findings of this study are available from the corresponding author upon reasonable request.
